# Bichromatic Photoassociation Spectroscopy for the Determination of Rotational Constants of Cs_2_ $0_{u}^{+}$ Long-Range State below the 6S_1/2_ + 6P_1/2_ Asymptote

**DOI:** 10.3390/molecules25173963

**Published:** 2020-08-31

**Authors:** Jizhou Wu, Jie Ma, Yuqing Li, Wenliang Liu, Peng Li, Vladimir B. Sovkov

**Affiliations:** 1 State Key Laboratory of Quantum Optics and Quantum Optics Devices, Institute of Laser Spectroscopy, Shanxi University, 92 Wucheng Road, Taiyuan 030006, China; wujz@sxu.edu.cn (J.W.); lyqing2006@sxu.edu.cn (Y.L.); liuwl@sxu.edu.cn (W.L.); 2 Collaborative Innovation Center of Extreme Optics, Shanxi University, 92 Wucheng Road, Taiyuan 030006, China; 3 College of Physics and Electronic Engineering, Shanxi University, 92 Wucheng Road, Taiyuan 030006, China; lip@sxu.edu.cn; 4 Department of Photonics, St. Petersburg State University, 7/9 Universitetskaya Nab., 199034 St. Petersburg, Russia

**Keywords:** ultracold molecule, diatomic molecules, molecular spectra, bichromatic photoassociation spectroscopy, long-range state, non-rigid-rotor model, rotational constants

## Abstract

This article demonstrates new observation of the high-resolution ro-vibrational bichromatic photoassociation spectra (BPAS) of Cs_2_ in the $0_{u}^{+}$ long-range state below the asymptotes 6S_1/2_ + 6P_1/2_. By combining with a modulation spectroscopic technique, precise references of the frequency differences have been engineered through the BPAS, with which the rotational constants of low-lying vibrational levels of the Cs_2_ $0_{u}^{+}$ long-range state have been accurately determined by fitting the frequency differences to the non-rigid-rotor model. The rotational constants for the newly observed seven ro-vibrational levels are summarized and disagreement for the level ῦ = 498 is clarified. The rotational constants of different vibrational levels demonstrate strong perturbations of the related energy structures. A simple analysis is performed and shows good agreement with experimental results.

## 1. Introduction

During trecent decades, quantum science and technologies have reached tremendous achievements benefiting from the ultracold atoms and molecules with a temperature extremely lower than 1 mK [1,2], under which the inter-particle collisions are fully quantum-mechanical and highly sensitive to long-range interactions. The straightforward preparation of an ultracold molecular sample is a challenging task, much harder than the production of ultracold atomic ensembles. The rich and complicated ro-vibrational energy level structure of molecules leads to a limited number of produced molecules and suppressed low phase-space densities obtained by direct cooling or laser cooling techniques, in which the temperature of molecules has not typically reached below ~µK [3]. Ideal (high-phase-space-density, low-temperature below 1 mK) gases of ultracold molecules can be produced from ultracold alkali atomic gases with indirect approaches such as photoassociation (PA) [4] or the magnetoassociation over a Feshbach resonance [5] followed by a Stimulated Raman Adiabatic Passage (STIRAP) [6] to deeply bound ground states [7]. Various robust and exquisite control techniques have enabled the ultracold molecules as promising candidates in extensive applications, such as precision measurement [8], cold-reaction chemistry [9], high-resolution spectroscopy [10], quantum information processing [11], and quantum simulation [12].

Due to its relatively low requirements for the initial temperature and density parameters of atomic samples, the PA has been proven to be a versatile and simple method to prepare weakly bound ultracold molecules at the micro-Kelvin range, both for homonuclear and heteronuclear molecules [4,13,14], which demonstrate van der Waals potentials proportional to 1/R^3^ or 1/R^6^ (where R is the inter-nuclear distance) in their excited molecular states at large internuclear separations. Also, ultracold molecules prepared by PA are characterized by small binding energies as the molecules are mostly formed in the near dissociation region. Furthermore, high-resolution Photoassociation Spectroscopy (PAS) greatly reduces the Doppler Effect, thereupon the detection accuracy can be enhanced to several kHz [15,16,17,18,19]. In this sense, the PAS is often advantageous in the detection of molecular ro-vibrational energy levels and hyperfine structures over traditional thermo-luminescence spectroscopy or fluorescence spectroscopy techniques [20]. PAS has enabled one to obtain information of molecular structures [21], to determine the long-range behaviour of the potential energy curves and molecular constants [22,23], and to measure the s-wave scattering lengths [4] as well as the time variations of fundamental physical constants [24].

Many experimental and theoretical studies were made on the long-range states of ultracold Cs_2_ molecules observed by the PA [25,26,27,28,29,30,31,32,33,34]. Firstly, the P. Pillet’s research group realized the formation of the ultracold Cs_2_ ground state molecules via the PA, obtained the PAS, and estimated the rotational constants for the vibrational levels of the $0_{u}^{+}$ and 1*_g_* states ranged from −5 cm^−1^ to −40 cm^−1^ relative to the Cs_2_ 6S_1/2_ + 6P_3/2_ dissociation limit [25]. M. Pichler and co-workers reported the spectroscopic studies of the Cs_2_ molecular excited states with the detuning of up to 56 cm^−1^ from the limits 6S_1/2_ + 6P_3/2_ [33] and 6S_1/2_ + 6P_1/2_ [34]. Recently, the spectroscopic detection detuning range has been extended to 70 cm^−1^ below the Cs_2_ 6S_1/2_ + 6P_3/2_ [21] asymptote by our group. Specifically, efforts have been made to further extend the PAS detection range, both to the high-lying molecular vibrational levels within the extremely dense states and to the lower-lying levels that are largely red detuned against the 6S_1/2_ + 6P_3/2_ [15,18] 6S_1/2_ + 6P_1/2_ [35] asymptotes. However, the rotational constants of the Cs_2_ $0_{u}^{+}$ levels below the dissociation limit 6*S*_1/2_ + 6*P*_1/2_, stemming from an *A*^1^$\Sigma_{u}^{+}$~*b*^3^Π_u_ complex in the Hund’s case (a) notation, so far have not been comprehensively investigated.

In this paper, we demonstrate accurate measurements of the rotational constants of the vibrational energy levels of the ultracold Cs_2_ molecules with high sensitivity. In our scheme, we have combined a new laser scan calibration scheme with modulation spectroscopy for the trap-loss fluorescence detection, thus leads to an appreciably improved signal-to-noise ratio. The scheme relies on calibrating frequency offsets within each laser scan. Partial output of the PA laser has passed through an acousto-optic modulator (AOM) to provide a calibration beam offset by 220 MHz. By mechanically switching from the main beam to the calibration beam during a laser frequency scan, a selected spectral feature can be scanned twice, namely bichromatic photoassociation spectroscopy (BPAS), with a known separation of 220 MHz that is used to calibrate the rest of the spectrum. The BPAS for specific vibrational levels in the Cs_2_ $0_{u}^{+}$ long-range state near the dissociation limit 6S_1/2_ + 6P_1/2_ are newly detected, with which the rotational constants are obtained with a high accuracy. The method proposed in this paper has benefitted a correction of the previously measured rotational constants for a specific ro-vibrational level. The simple analysis demonstrates the strong perturbation effect of the $0_{u}^{+}$ state. Our results provide a framework for future works exploring the precise potential energy curves of the long-range states. 

## 2. Experimental Details

**Figure 1:**
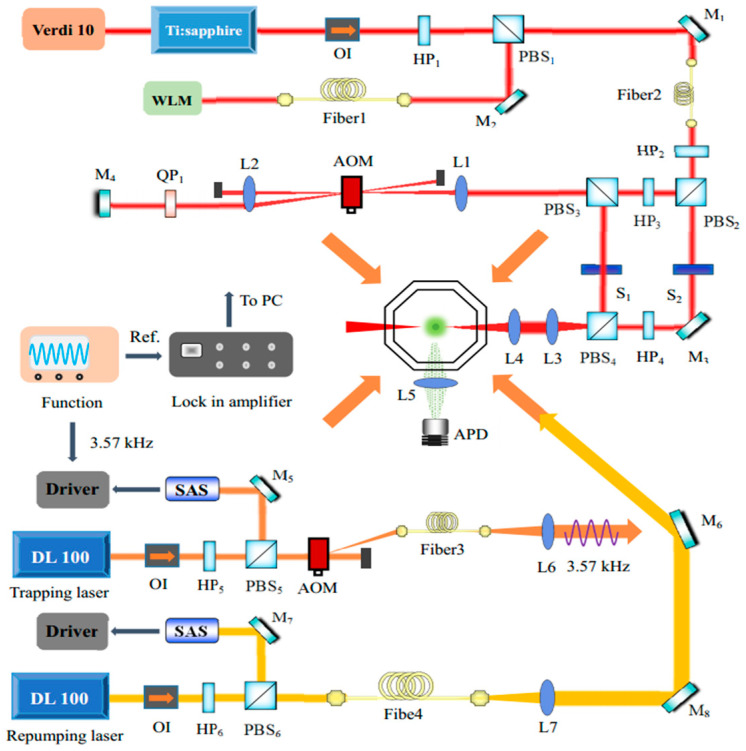
Experiment setup: OI: optical isolator; H: halfwave plate; PBS: polarization beam splitter; AOM: acousto-optic modulator; L: lens; M: high reflective mirror; S: shutter; Q: quarter-wave plate; APD: avalanche photodiode; SAS: saturation absorption spectroscopy; PC: computer.

The experimental scheme for the measurement of the BPAS of ultracold Cs_2_ molecules is similar to the one reported in Ref. [18], as demonstrated schematically in Figure 1. The ultracold cesium atoms were prepared by the magneto-optical-trap (MOT) technology in a vacuum chamber with a background pressure of 3 × 10^−7^ Pa. The trapping light and the re-pumping light were provided by two semiconductor lasers, whose frequencies were stabilized with the standard saturated-absorption technology. The trapping laser power of totally ~39 mW was equally divided into three pairs of beams, which were transmitted through the optical fiber into the center of the cell with mutually opposite circular polarizations. A pair of anti-Helmholtz coils generated a quadrupole magnetic field along the axis of the coils with a gradient of ~16 Gauss/cm to trap the cooled ultracold cesium atoms. The repumping laser beam with a power of ~5 mW directly illuminated the MOT after the collimation of the fiber. The temperature of the ultracold cesium atomic cloud was ~200 µK, measured by the time-of-flight method. The number of atoms was ~5.1 × 10^7^ with a characteristic diameter of the cloud of ~800 µm. The cesium MOT was monitored in real time by a Charge Coupled Device (CCD) camera (MintronMTV-1881EX).

A continuous tunable Ti:sapphire laser (MBR110, linewidth ~0.1 MHz, power ~1.2 W), which was pumped by a diode-pumped solid-state laser (Verdi 10, ~10 W) with an output at 532 nm, served as the PA laser. In the experiment, a commercial wavelength meter (High Finesse-Angstrom WS/7R, absolute accuracy ~60 MHz) was used to monitor and measure the absolute PA laser frequency. The cesium atomic hyperfine resonance transition, 6S_1/2_ (F = 3) → 6P_1/2_ (F′ = 4), corresponding to the wavenumber of ν_D_ = 11178.151 cm^−1^, was referred to the calibration of the wavelength meter. The PA acts on two colliding ultracold atoms resonantly absorbing a photon with frequency ν to form a weakly bound excited state molecule, which spontaneously decays (lifetime ~15 ns) into the ground state or de-excites to two free atoms escaping from the MOT. Both decaying channels lead to the instantaneous loss of the ultracold atoms from the MOT. The PAS directly illustrates the number of the produced excited molecules by recording the variation of the fluorescence of the MOT, i.e., the trap loss, as a function of ν. The trap loss detection method is typically advantageous over the ionization method by providing both the frequency positions of the PA resonances and the transitional strengths, which is important for studying the PA process and optimizing the energy schemes in the STIRAP experiments but not always available by the ionization method. The fluorescence was collected by a convex lens and detected by a silicon avalanche photodiode (APD S3884) with two pieces of 850 nm band-pass filters.

The traditional direct fluorescence spectroscopic technology usually suffered from a poor signal-to-noise ratio and low detection sensitivity due to the presence of various stray light and noise in the MOT. A lock-in method, based on modulating the fluorescence of the cesium atoms, was used to improve the detection sensitivity of the trap loss spectroscopy in our experiment. The trapping laser frequency was modulated by an auxiliary signal with a frequency of 3.57 kHz, which was also used to stabilize the laser’s frequency. The modulated fluorescence was collected through an APD, demodulated by a lock-in amplifier (SR830), and recorded by a computer.

On the other hand, the BPAS technology constructs the accurate frequency reference standards and measures the frequency intervals of neighboring rotational levels with the improved precision benefitting from the modulation technique. In this way, the rotational constants of the ultracold Cs_2_ molecules were obtained. The output of the PA laser was divided into two beams by passing through a half-wave plate (HP2) and a polarization prism beam splitter (PBS2). The frequency of one beam (Beam II) was reduced (shifted downwards) from the one of the main beam (Beam I) by Δν_0_ = 220 MHz via an AOM-based double-pass configuration (the carrier frequency of the AOM was set as 110 MHz).

## 3. Results and Discussion

**Figure 2:**
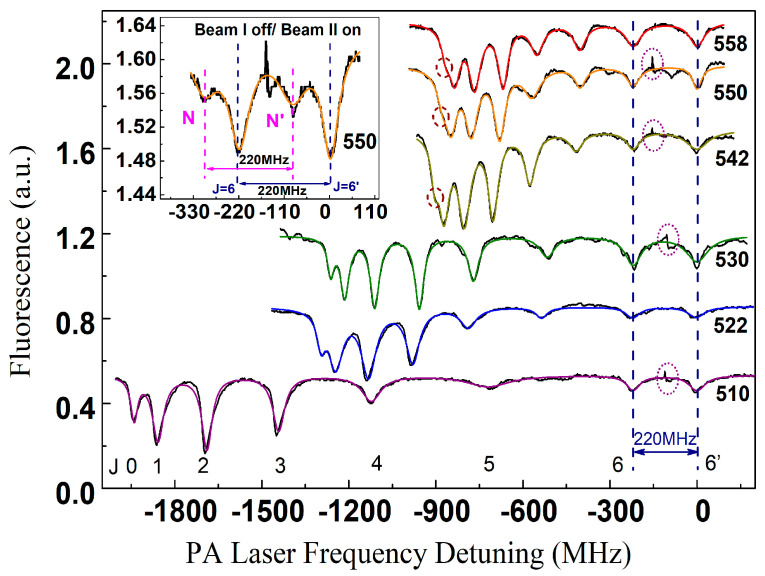
Six typical BPAS of the vibrational levels ῦ = 558, 550, 542, 530, 522, 510, of ultracold Cs_2_ $0_{u}^{+}$ (6S_1/2_ + 6P_1/2_) long-range state. The intensities of the PA laser beams (I and II) were 56 W/cm^2^ and kept unchanged. The Ti:sapphire laser slowly scanned with stringent linearity. The colorful curves are the multipeak Lorentzian fits. The *J* = 0 rotational progressions for the levels with small binding energies, which are usually quite close to the *J* = 1 ones, are denoted as circles. The influences on the fluorescence due to the switch of the shutters are shown in the ellipses. The case for ῦ = 550 progression is zoomed in the inset, where the *N* and *J* = 6 indicate the spectrum produced by the Beam I scans, while *N*′ and *J*′ = 6 are for the Beam II scans. The *N* feature could stem from the strong perturbation [36] from the neighboring $0_{g}^{-}$ and 1*_g_* states. Actually, in previous studies on the Cs_2_ $0_{g}^{-}$ (6S_1/2_ + 6P_1/2_) long-range state [35], we had also observed the same phenomena. The separation between the *N* and *N*′ features is Δν_0_ = 220 MHz, as expected.

In the experiment, the typical bichromatic PA spectrum was recorded by alternately alternately shining the Beam I and Beam II from the PA laser on the MOT. The ro-vibrational PA spectrum for the rotational quantum number from *J* = 0 to 6 was firstly obtained by the trap loss that was induced by the Beam I. As the whole loss spectrum for *J* = 6 was acquired, Beam I was switched off. Then Beam II simultaneously switched on, which interacted with the MOT and, in turn, induced an atomic loss. In other words, the *J* = 6 level was first scanned by Beam I and then by Beam II. The losses induced by Beam II was denoted as J’ in order to not cause confusion. Beams I and II were set with the same intensity and well overlapped in space before interacting with the cesium MOT. Two shutters were used to independently control the two beams. The switching of the two beams was controlled by the shutters in a much shorter time (<3 ms) than the loading time (~8 s). Therefore, it did not cause significant changes in the fluorescence, except the spike in Figure 2 inset. It should be noted that the BPAS scheme strictly relies on the high linearity of the scanning rate of the PA laser, which was monitored in real time using the wavelength meter (WS/7R).

Figure 2 demonstrates the BPAS of the vibrational levels ῦ = 558, 550, 542, 530, 522, 510, which are red detuned from the Cs_2_ 6S_1/2_ + 6P_1/2_ dissociation limit. The ῦ designation is for an ordinal number of a level starting from ῦ = 0 at the bottom of the *A*^1^$\Sigma_{u}^{+}$~*b*^3^Π_u_ mixing [28,29,30]. It is worth noting that the notation is the same as that of Ref. [31], while other reports [31,32,34] usually using the notation of [*v_D_*] − *v*, where [*v_D_*] = 710, is the virtual vibrational quantum number at the dissociation limit of the near-dissociation theory. An estimate of *v_D_* differs in different analyses [31,32,33] and depends on its interpretation (which levels are counted). Further on, we used our latest result in Ref. [31]: [*v_D_*] = 710 with all the coupled states of the symmetry $0_{u}^{+}$ below 6S_1/2_ + 6P_1/2_ counted. We would also like to emphasize that in the context of the present article, this value is only needed for the level identifications and does not influence any other physically significant consequences. The binding energies *T_v_* of specific levels are listed in Table 1. *T_v_* = ν − ν_D_, where the ν is the PA laser frequency of a specific energy levels. The spectra show similar intensity undulations on account of the Franck-Condon factors (FCFs), i.e., the photoassociation transitions between the initial scattering state and the final ro-vibrational levels in the long-range $0_{u}^{+}$ states. The *J* = 0–6 spectra possess high resolution. The intensity of the spectral lines corresponding to these rotational energy levels gradually increases from *J* = 0 to the maximum value at *J* = 2, then gradually decreases to *J* = 6. The rotational progressions of higher-order terms stem from the higher orbital angular momentum contributors (*p*-wave or *d*-wave) than the *s*-wave component.

The rotational level *J* = 6 was scanned by the Beam II, which was denoted as *J*′ = 6, after the Beam I scanned it. The two beams were separated by a fixed frequency difference Δν_0_ = 220 MHz, which was set via the AOM with an accuracy of ~kHz. To shield the influences of laser-induced frequency shifts [37] of individual levels that could lead to inaccuracy of the measurements, the two beams were adjusted within the same intensities. Thus, the frequency interval between *J* = 6 and *J*′ = 6 was exactly Δν_0_ = 220 MHz, i.e., the fixed frequency difference between the Beam I and Beam II, which was used as a precise reference to calibrate the frequency separations Δν*_J_* between neighboring rotational levels in the BPAS. By fitting the data of Δν*_J_* to the rotor model [38], the rotational constants of specific vibrational levels, which are listed in Table 1, were obtained with high sensitivity. Compared to previous studies [18,34], the signal to noise ratio (SNR) of the current investigation was improved to ~60, and the improved sensitivity has led to an enhanced accuracy of the measurements.

**Figure 3:**
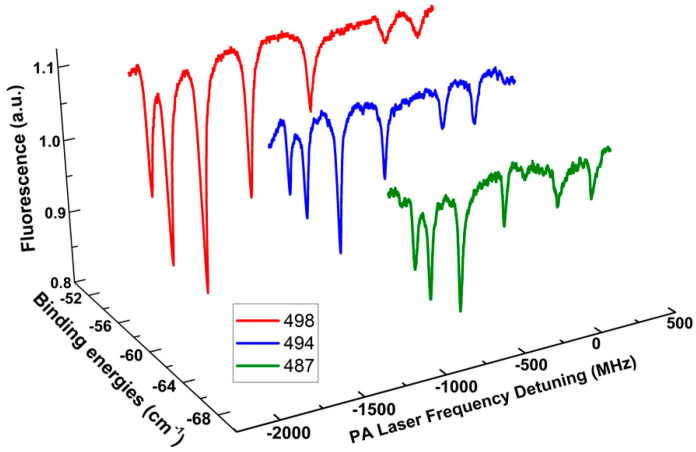
BPAS of the vibrational levels ῦ = 498, 494 and 487 of ultracold Cs_2_ $0_{u}^{+}$ (6S_1/2_ + 6P_1/2_) long-range state. The spectroscopy data have been extended to a red detuning of nearly ~70 cm^−1^ below the 6S_1/2_ + 6P_1/2_ dissociation limit.

**Figure 4:**
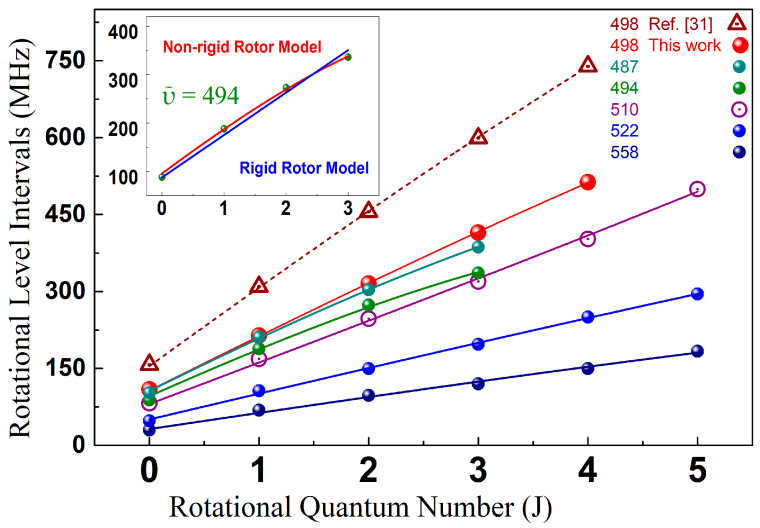
Relationship between *J* and the frequency rotational intervals Δν for specific ro-vibrational levels in the Cs_2_ $0_{u}^{+}$ state. Experimental data, shown in colorful symbols were fitted by the nonlinear non-rigid-rotator model. The former incorrect calibrated values for ῦ = 498 [31], which were misprinted as ῦ = 499 in the reference, are also presented as triangles for comparison.

**Figure 5:**
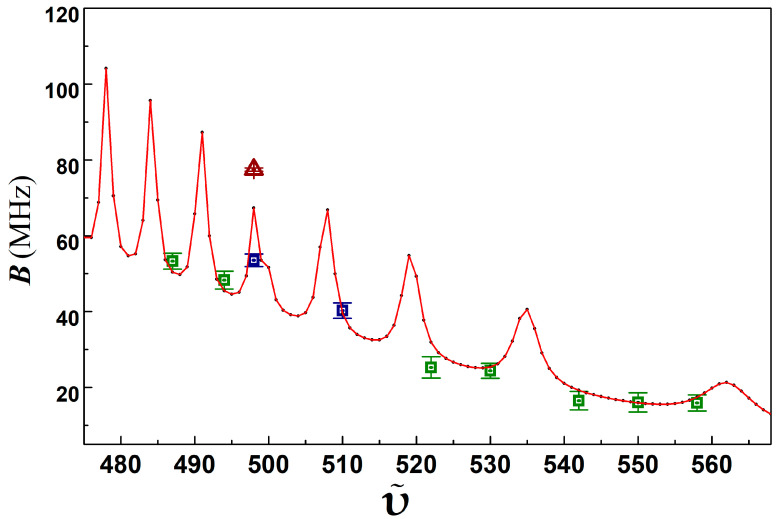
Rotational constants Bv of the rovibrational levels (ῦ) in Cs_2_ $0_{u}^{+}$ state below the dissociation limit 6S_1/2_ +6P_1/2_. Triangle is the value reported in Ref. [31], other newly observed values are in green squares. The blue squares for ῦ = 498 and 510, which were observed previously, have also been measured and presented for comparison. The black dots are the theoretical rotational constants, computed in a two channel quantum mechanical simulation [31]. The solid red curve is the connection of the dots. The newly observed values are in good consistence with the theoretical curve.

In Figure 3, we show the case for ῦ = 498, i.e., [*v_D_*]-*v* = 217 in Ref. [34]. It should be noted that in the previous studies [31], there was a misprint in Table 1, i.e., the ro-vibrational level ῦ = 499 should be 498, whose rotational constant was reported as 77.48 ± 0.42 MHz. This value looks excessively large, as Figure 4 and Figure 5 show, compared to the value reported in Ref. [34]. We systemically tested the former experimental data as we carried out in Ref. [31] and found that the data for that level was wrongly processed with mistaken Δν_0_ = 215 MHz instead of 220 MHz. In the current study, we have rescanned this level again and processed the data carefully and found that the corrected rotational constant for ῦ = 498 should be 53.57 ± 1.67 MHz. The BPAS of this level has been demonstrated in Figure 3. The influence from the gain of the accuracy of this level on the molecular potential energy curve, which is not the main theme of the current study, will be estimated by compared to the analysis in Ref. [36]. It is worth mentioning that we have extended the investigations to ῦ = 494 and 487, whose BPAS have also been exhibited in Figure 3. The extension of the BPAS, as well as the *B_v_* constant estimates, of the $0_{u}^{+}$ state far from the dissociation limit 6S_1/2_ + 6P_1/2_, will be performed in future studies. Signals for the rotational progressions with higher than *J* = 5, 6 with lower signal amplitudes are not considered in the present work.

The non-linear relationship between frequency intervals Δν*_J_* and *J* for different vibrational levels in the Cs_2_ $0_{u}^{+}$ state below the 6S_1/2_ + 6P_1/2_ dissociation limit, which originated from the molecular long-range interaction between the two constituent atoms, is demonstrated in Figure 4. When fitting the data, we have considered the rigid-rotor model and the non-rigid-rotor model. The rigid rotor model can be considered to be the 0th order description of a molecule rotational motion, which neglects the coupling to the vibrational motion and the fact that rotating molecules do not behave as rigid-body. Thus, the rigid rotor model is often employed to describe low-*J* transitions of weakly bound molecular complexes. On the other hand, the non-rigidity of the molecular bonds and the vibrational-rotational interaction makes centrifugal distortion non-negligible. In particular, the ultracold Cs_2_ molecule formed by the photoassociation is mainly located in the long-range state with a very large inter-nuclear distance. Therefore, the centrifugal distortion effect are important for the description of rotational transitions at increasing *J* quantum numbers, where the larger rotational energy causes a measurable distortion of the molecule and hence a departure of the energy levels from the rigid-rotor pattern, as the inset of Figure 4 shown. In this way, the fittings of Δν*_J_* were performed by using the molecular non-rigid-rotor rotor model [38], the results are shown as curves of different colors in Figure 4. This model implements the following equation:

*E_J_*/*hc* = *B_v_J* (*J* + 1) − *D_v_J^2^* (*J* + 1)^2^ (1)

where *E_J_* is the rotational energy, *h* is the Planck constant, *c* is the speed of light, *B_v_* is the rotational constant, and *D_v_* is the centrifugal distortion constant. The formula of adjacent frequency interval used for fitting is as follows:

∆*ν_J_* = (*E*_*J* + 1_ − *E_J_*)/*hc* = *2B_v_*(*J* + 1) −4*D_v_* (*J* + 1)^3^ (2)

The values of *B_v_* and *D_v_* can thus be obtained by fitting the accurate rotation level intervals to Equation (2). As *D_v_* is three orders smaller than *B_v_*, it usually does not play an important role in the analysis; thereupon we only report the *B_v_* value. These *B_v_* and their estimated inaccuracies for specific vibrational levels are listed in Table 1. The uncertainty mainly came from the determination of the resonant peak position and the statistical error of fitting. In this analysis, the positions of the lines are taken as given by the location of their maximum. However, lines profiles are complicated, influenced by hyperfine interactions, natural linewidth, partial wave dependency, etc. [21,39]. Therefore, we did not try here to estimate the deperturbed line positions. Consequently, the rotational constants, extract from these line positions, can only be taken rough estimations of the real values and do not have any spectroscopic accuracy. The BPAS scheme and the non-rigid-rotor model enabled the accurate measurements of the molecular rotational constants.

Figure 5 illustrates the relationship between *B_v_* values for the ro-vibrational levels of the Cs_2_ $0_{u}^{+}$ 6S_1/2_ + 6P_1/2_ long-range state and the vibrational quantum numbers ῦ. The corrected value made the high-lying vibrational levels more consistent with those for the low-lying counterpart. The current investigation reaches more accurate results than previous studies. Similar behavior of the rotational constant in the $0_{u}^{+}$ state of ^85^Rb_2_ [40] was also reported. The fluctuations of *B_v_* values indicate a strong spin-orbit coupling between the *A*^1^$\Sigma_{u}^{+}$~*b*^3^Π_u_ mixing electronic states. Meanwhile, we found that the rotational constants for the Cs_2_ $0_{g}^{-}$ state below the asymptote 6S_1/2_ + 6P_1/2_, demonstrating a smooth variation [35], which can also be interpreted by a semi-experimental method [41], do not show the same behavior as that for the $0_{u}^{+}$ state. It would be interesting to jointly analysis the different behaviors of the two long-range states through future experimental measurements and theoretical models [42] to unveil the physical mechanisms.

## 4. Conclusions

In conclusion, a BPAS scheme was introduced to accurately measure the rotational constants of the ultracold Cs_2_ $0_{u}^{+}$ long-range state below the 6S_1/2_ + 6P_1/2_ dissociation limit with high sensitivity and high resolution. A series of the BPAS spectra of Cs_2_ were demonstrated. A frequency reference standard was built in combination with the modulation spectroscopic technology to acquire the frequency intervals of adjacent rotational energy levels without using a high-precision and expensive wavelength meter. The SNR as well as the sensitivity of the current research was greatly enhanced. By fitting the data to a non-rigid rotor model, which proved to be statistically significant vs. the rigid rotor one, the rotational constants of seven newly observed ro-vibrational levels were obtained. The proposed simple and robust scheme can be versatilely used to study the behaviors of other long range states of ultracold molecular species.

## Figures and Tables

**Table 1:** Rotational constants *B_v_* and centrifugal distortion constants *D_v_* of 7 low-lying ro-vibrational levels in the $0_{u}^{+}$ state below the Cs_2_ dissociation limit 6S_1/2_ + 6P_1/2_. The numbering of the levels [*v_D_*]-*v* from Ref. [34] has been corrected [32] in the current study and has been assigned as ῦ, as listed in the table. The binding energies *T_v_*, are related to *J* = 2 of each level, whose PA laser frequencies have also been provided as ν. The ῦ = 510 case is listed for reference, ῦ = 498 has been corrected compared to former studies [31].

ῦ	[*v_D_*]-*v* [32]	[*v_D_*]-*v* [34]	*T_v_*, *J* = 2 (cm^−1^)	*B_v_* (δ*B_v_*) (MHz)	*D_v_* (δ*D_v_*) (MHz)	ν, *J* = 2 (cm^−1^)
558	152	157	−7.612	15.92 (2.12)	0.04 (0.02)	11,170.539
550	160	165	−10.126	16.09 (2.53)	0.05 (0.04)	11,168.025
542	168	173	−13.480	16.52 (2.42)	0.04 (0.02)	11,164.671
530	180	185	−20.046	24.41 (1.98)	0.03 (0.02)	11,158.105
522	188	193	−26.120	25.29 (2.82)	0.06 (0.03)	11,152.031
510	200	205	−37.312	40.25 (2.02)	0.07 (0.05)	11,140.839
498	212	217	−51.488	53.57 (1.67)	0.05 (0.02)	11,126.663
494	216	221	−57.617	48.29 (2.34)	0.18 (0.04)	11,120.534
487	223	228	−68.854	53.34 (2.09)	0.15 (0.03)	11,109.297
